# Heart Rate Variability and Vagus Nerve Stimulation in Epilepsy Patients

**DOI:** 10.1515/tnsci-2019-0036

**Published:** 2019-08-24

**Authors:** Victor Constantinescu, Daniela Matei, Irina Constantinescu, Dan Iulian Cuciureanu

**Affiliations:** 1 Neurology Department, Faculty of Medicine, University of Medicine and Pharmacy Iasi, Iasi Romania; 2 Department of Biomedical Sciences, Faculty of Medical Bioengineering, University of Medicine and Pharmacy Iasi, Iasi Romania; 3 Neurology Department, Regional Hospital of Orléans, Orléans France

**Keywords:** cardiac autonomic modulation, sympathovagal balance, sympathetic and parasympathetic activation tests, heart rate variability, multiple trigonometric regressive spectral analysis, vagus nerve stimulation, drug-resistant epilepsy

## Abstract

**Background:**

Vagus nerve stimulation (VNS) exerts a cortical modulating effect through its diffuse projections, especially involving cerebral structures related to autonomic regulation. The influence of VNS on cardiovascular autonomic function in drug-resistant epilepsy patients is still debated. We aimed to evaluate the impact of VNS on cardiovascular autonomic function in drug-resistant epilepsy patients, after three months of neurostimulation, using the heart rate variability (HRV) analysis.

**Methodology:**

Multiple Trigonometric Regressive Spectral analysis enables a precise assessment of the autonomic control on the heart rate. We evaluated time and frequency-domain HRV parameters in resting condition and during sympathetic and parasympathetic activation tests in five epilepsy patients who underwent VNS procedure.

**Results:**

We found appropriate cardiac autonomic responses to sympathetic and parasympathetic activation tests, described by RMSSD, pNN50, HF and LF/HF dynamics after three months of VNS. ON period of the neurostimulation may generate a transient vagal activation reflected on heart rate and RMSSD values, as observed in one of our cases.

**Conclusion:**

VNS therapy in epilepsy patients seems not to disrupt the cardiac autonomic function. HRV represents a useful tool in evaluating autonomic activity. More extensive studies are needed to further explore cardiac autonomic response after neurostimulation.

## Introduction

Epilepsy affects approximately 65 million people worldwide [1]. Although therapy has substantially developed, about a third of patients remain resistant to drug treatment [1]. This leads to high mortality and morbidity [2, 3]. Prevention measures and recognition of modifiable risk factors may reduce epilepsy mortality.

Patients with drug-resistant epilepsy, defined as seizure occurrence despite judicious association of two or more antiepileptic drugs over a minimum of 12 months period [4], may benefit from epilepsy surgery. Currently, this is performed in a small subset of drug-resistant patients. Vagus nerve stimulation (VNS) represents an adjuvant treatment for medically refractory partial-onset seizures in adults and adolescents [5]. VNS consists of chronic intermittent electrical stimulation of the vagus nerve, delivered by a programmable pulse generator [5]. VNS may represent an earlier stage option in treating pharmacoresistant epilepsy, with positive long-term effects [6, 7], reducing the frequency of seizures and ameliorating the quality of the interictal period [7].

VNS exerts through its diffuse projection via the nucleus of the solitary tract, or the reticular system, a cortical modulating effect, especially involving cerebral structures related to autonomic regulation, such as thalamus, amygdala or prefrontal region [8]. The impact of VNS on cardiovascular autonomic function in drug-resistant epilepsy patients remains a controversial subject and in need of further studies [9].

Heart rate variability (HRV) represents a simple and non-invasive method to evaluate the sympathovagal balance, outlining the cardiac ability to adapt to hemodynamic and pathological conditions. Sympathetic hyperactivation and reduced cardiac vagal modulation associated with low HRV determines higher risk of cardiac arrhythmia and sudden death [10].

Rüdiger et al. proposed a novel algorithm to detect physiological oscillations of the heart rate (HR) based on RR intervals measurements – multiple trigonometric regressive spectral (MTRS) analysis [11]. HRV analysis in epilepsy provides essential information about the risk of sudden death by cardiac arrhythmias in these patients [12].

The aim of this research is to evaluate the impact of the VNS on cardiovascular autonomic function, through sympathetic and parasympathetic activation tests, after three months of neurostimulation in drug-resistant epilepsy patients, using MTRS analysis of the HRV.

## Methods

ECG recordings of the first five drug-resistant epilepsy patients from our department who underwent left laterocervical VNS procedures were analyzed using MTRS software.

None of the five patients underwent epilepsy surgery and had no cardiovascular comorbidities, as well as no cardiovascular medication. No changes in the antiepileptic medication were performed three months prior to our first evaluation and between the two HRV tests.

ECG was registered before the VNS procedure and at least three months after the

implantation of the electrode. ECG recordings were performed during ON and OFF time period of VNS stimulation.

Blood tests including electrolytes, hepatic and renal function were within the normal range for all patients in both evaluations.

All patients were informed about the study protocol and gave written consent in accordance with ethical principles. The study was carried out in accordance with the Helsinki Declaration.

We applied a standardized protocol including resting state and subsequently four autonomic activation tests, each test entailing a five minute ECG recording. There was a HR stabilization before starting the evaluation. Tests were performed at the same time range (4-6 PM), after 30 minutes of resting position in clinostatism, at a constant temperature of 22ᴼC, in a quiet room, in the absence of sounds or human voices, without prior physical effort or ingestion of caffeinated or alcoholic beverages 24 hours before the evaluation. All patients were asked to empty the bladder before starting the evaluation. Moreover, the tests were performed at least three hours after lunch in order to avoid gastric distension. Two sympathetic activation tests were performed: a maximal voluntary isometric contraction of the fist, using a dynamometer – “hand-grip” test and a three-minute standing test. Two parasympathetic activation tests were considered: “deep breathing” test, consisting of six complete cycles of deep inhale and exhale over 60 seconds, with timing, 10 seconds for each cycle, and Valsalva maneuver. The test sequence was standardized: resting state, deep breathing test, hand-grip test, standing test and Valsalva maneuver for all patients.

BIOPAC^®^ acquisition system was used for data collection and analysis. BIOPAC^®^ represents an integrated hardware and software system that converts biologic parameters like HR to numeric data. AcqKnowledge software version 3.9.1.6 was used for refining the recorded data. Data processing was done using MTRS software version 7.3.2.0 (University Hospital, Center for Clinical Neuroscience, Dresden, Germany). Before each analysis, a manual data correction of ECG artifacts was carried out.

The dynamic assessment of HRV by MTRS analysis allows a precise evaluation of cardiovascular modulation under different conditions. This software assesses the HRV time-domain and frequency-domain parameters, based on the trigonometric regressive analysis [13]. The HRV parameters are often analyzed by Fourier Transform. The mathematical approach using trigonometric regressions excluded the RR intervals equidistance issue, arising with the method of Fourier Transform [11].

MTRS works with two data segments, a global and a local data segment. The global data segment considered was the length of the ECG recording (5 minutes) and the local data segment was set - 30 seconds. The analysis is performed in all local data segments, until the end of the global data segment. All oscillations of the biosignals (RR intervals) are analyzed using a trigonometric function of the parameters A (amplitude), ω (frequency), and ϕ (phase shift) [13]. For each recording, the same settings of the local data segment were applied: a minimum variance reduction of 1%, a shift of the local data segment of 1 RR interval and Delta frequency 0.006 Hz. These settings remained unchanged for all patients. Considering non-stationary signals analysis, a trend correction over every local data segment was performed automatically in each local time window before the final analysis.

Heart rhythm oscillations may be categorized into four primary frequency bands: Ultra Low Frequency (ULF), Very Low Frequency (VLF), Low Frequency (LF) and High Frequency (HF). Respiration modulates vagal activity and contributes to the HF component of the spectra, ranging from 0.15Hz to 0.4Hz [10]. Recent clinical and animal studies concluded that the LF component (0,04-0,15 Hz) of the HRV probably reveals a complex and not easily discernible mix of sympathetic, parasympathetic, and other factors interaction, and does not accurately reflect changes in the sympathetic activity [14], as initially presumed. Other studies suggested that LF component reflects the baroreflex-mediated, phasic changes in cardiovagal and sympathetic noradrenergic outflows [15]. As a consequence, the physiological basis for LF/HF is difficult to discern [14]. The LF and HF values may also be calculated in normalized units (LFnu, HFnu), defining the relative values of each frequency spectrum reported to the total spectral power, from which the VLF component was excluded from the calculation.

The following time-domain parameters were considered: pNN50 (the proportion of pairs of successive RR intervals that differ by more than 50 ms to the total number of NN intervals) and RMSSD (the square root of the mean squared differences of successive NN intervals). These two parameters reflect the parasympathetic influence on the cardiac rhythm [10].

HRV is significantly correlated with an average HR, dependent on the influence of autonomic nervous system activity [10] and also mathematically determined (i.e., the inverse non-linear relationships between HR and RR interval) [16]. Therefore it is needed to distinguish if the clinical significance of HRV comes from the variability or from HR [17]. If the variability of a slow HR is compared with a fast HR (based on the fluctuations of RR intervals), greater HRV in patients with former than with the latter can be obtained [18]. To reduce this influence, we corrected the HRV for the average HR. The correction consisted of dividing or multiplying standard HRV indices by the power of two of their corresponding mean RR interval (mRR) [16].

This correction procedure does not remove any information about the signal’s oscillations but only makes the oscillations relative to the signal’s average value [18]. This allows the comparison of HRV among patients with different average HRs [19, 20].

HRV is also influenced by the respiratory rate (RespRate). Therefore, alterations in these parameters may impose changes in HRV [16, 21]. A decrease in respiratory frequency generally corresponds with a lengthening of the heart period [22, 23]. RespRate may be calculated from ECG recordings according to Sinnecker et al. [24].

The analysis was performed using GraphPad Prism software version 8.1.0. For the statistical analysis of the data, having into consideration the small sample size, series normalization was very difficult. Wilcoxon matched-pairs tests were applied to compare the parameters of the analyzed series. Spearman’s rank correlation coefficient (rs) was used to assess the relationship between variables, and Student’s t-test or non-parametric Mann–Whitney test were employed to determine differences. The significance level (p-value) was considered to be 0.05 (5%).

## Results

Clinical symptoms, type of epilepsy, age of onset, cerebral MRI findings and current treatment are depicted in table 1. Patients 1 and 3 were seizure-free in last month before the second HRV evaluation, patient 2 had no seizure in the last three weeks before the second HRV evaluation and patient 4 had no change in seizure frequency after three months of neurostimulation.

**Table 1:** Patients description

Patients Age/gender	Clinical symptoms	Age of onset	Type of epilepsy	Brain MRI	Current treatment
Patient 1	rotatory vertigo, breathing difficulties,	8	focal epilepsy	no epilepsy-related	Lamotrigine,
33/female	dreamy state, facial rush, generalization		(left anterior temporal), secondarily generalized seizures	abnormalities	Levetiracetam and Oxcarbazepine
Patient 2	abnormal sensation of retrosternal	6	focal epilepsy	no epilepsy-related	Valproic acid and
34/female	pain, nausea, dyspnea, burning “heat” restricted in the perioral area, anarthria		(left insular epilepsy)”	abnormalities	Levetiracetam
Patient 3	retrosternal ascending “heat”,	4	focal epilepsy	left insular atrophy with	Valproate and
31/male	hypersalivation and post-ictal psychomotor agitation with hetero- aggressive behavior		(left insular epilepsy) with secondarily generalized seizures	frontoparietal extension	Oxcarbazepine
Patient 4	vertigo, sweating and motor unilateral	8	multifocal epilepsy with	Parietal and occipital	Lamotrigine,
29/female	symptoms, motor aphasia and generalization		secondarily generalized seizures	gyration abnormalities	Levetiracetam and Carbamazepine
Patient 5	rotatory vertigo, facial flush, sense of	22	focal epilepsy (right insular	no epilepsy-related	Levetiracetam and
34/female	unreality and generalization		epilepsy) with secondarily generalized seizures	abnormalities	Oxcarbazepine

Seizure frequency decreased for patient 3 in the last month before the second HRV evaluation, during VNS therapy. VNS parameters of the five patients are summarized in table 2.

**Table 2:** VNS parameters of the five patients

VNS parameters	Patient 1	Patient 2	Patient 3	Patient 4	Patient 5
Normal mode – Output current	2mA	1.5mA	1mA	2mA	2mA
Normal mode – Frequency	30Hz	30Hz	30Hz	30Hz	30Hz
Normal mode – Pulse Width	500 μsec	500 μsec	500 μsec	500 μsec	500 μsec
Normal mode – Duty Cycle	10%	10%	10%	10%	10%
Normal mode – ON time	30sec	30sec	30sec	30sec	30sec
Normal mode – OFF time	5min	5min	5min	5min	5min

Our ECG recordings did not reveal bradycardia, tachycardia or other cardiac arrhythmias in resting state or during challenge for all five patients. MTRS analysis of the ECG recordings from the five patients during resting state and autonomic activation tests provided time-domain (table 3) and frequency-domain parameters. To reduce the influence of HR on the HRV, the parameters that revealed a negative relationship with HR, as RMSSD, pNN50 and HF were divided by mRR squared to become HR independent. The parameter positively related to HRV (LF/HF ratio) was multiplied by mRR squared [16, 19].

**Table 3:** Time-domain parameters RMSSD and pNN50 provided by MTRS analysis

RMSSD (ms) / mRR (ms)					
	RS	DB	HG	ST	VA
Patient 1	16.92 / 672.04	17.92 / 654.24	15.43 / 659.62	14.23 / 648.60	16.86 / 650.82
	14.96 / 659.72	16.02 / 675.86	13.92 / 675.74	10.55 / 586.11	15.33 / 707.65
Patient 2	32.60 / 784.96	31.92 / 766.67	26.32 / 767.27	22.13 / 757.84	50.74 /762.54
	56.97 / 882.40	54.84 / 822.0	35.99 / 766.96	31.57 / 816.03	68.51/ 897.17
Patient 3	30.60 / 814.14	30.66 / 814.73	19.62 / 776.57	21.78 / 760.08	25.01 / 802.67
	52.71 / 807.33	51.86 / 813.28	49.29 / 713.58	50.15 / 710.23	51.86 / 801.46
Patient 4	13.95 / 753.19	15.11 / 790.86	13.07 / 734.40	9.70 / 752.70	16.55 / 790.91
	13.47 / 809.55	15.47 / 825.38	12.77 / 784.77	17.94 / 799.35	21.64 / 822.14
Patient 5	16.04 / 666.29	19.85 / 660.71	17.96 / 674.05	14.40 / 641.75	21.37 / 674.19
	19.53 / 660.71	24.59 / 727.13	22.47 / 743.25	16.64 / 678.53	28.18 / 763.04

pNN50 (%) / mRR (ms)					
Patient 1	0 / 672.04	0 / 654.24	0 / 659.62	0 / 648.60	0 / 650.82
	0.50 / 659.72	0.42 / 675.86	0 / 675.74	0 / 586.11	0.65 / 707.65
Patient 2	10 / 784.96	8.02 / 766.67	4.5 / 767.27	3.14 / 757.84	11.11 / 762.54
	28.03 / 882.40	15.31 / 822.0	10.95 / 766.96	12.59 / 816.03	28.07 / 897.17
Patient 3	7.08 / 814.14	7.17 / 814.73	2.08 / 776.57	3.94 / 760.08	5.99 / 802.67
	23.99 / 807.33	26.14 / 813.28	12.26 / 713.58	12.50 / 710.23	27.33 / 801.46
Patient 4	0.75 / 753.19	1.11 / 790.86	0.58 / 734.40	0 / 752.70	1.90 / 790.91
	0 /809.55	1.20 / 825.38	0.48 / 784.77	0.39 / 799.35	3.68 / 822.14
Patient 5	0.70 / 666.29	3.44 / 660.71	0.23 / 674.05	0.88 / 641.75	3.71 / 674.19
	0.75 / 660.71	4.63 / 727.13	2.08 / 743.25	0.31 / 678.53	5.04 / 763.04

Legend: Test 1 (HRV parameter/mRR), Test 2 (HRV parameter/mRR); RS=resting state; DB=deep breathing test; HG=hand-grip test; ST=standing test; VA=Valsalva maneuver mRR=mean RR interval.

In order to analyze the relationship before and after correction of the parameters, we performed Wilcoxon matched-pairs signed rank test. Spearman’s rank correlation coefficient “rs”, calculated comparing values before and after normalization, is displayed in table 4 for each patient.

**Table 4:** Wilcoxon matched-pairs signed rank test before and after correction for HRV parameters

Patient	RMSSD			pNN50			HF			LF/HF	
Patient 1	rs=0.87	p=0.0008	rs=0.99		p=0.0014	rs=0.92		p=0.0002	rs=0.96		p<0.0001
Patient 2	rs=0.90	p=0.0004	rs=0.97		p<0.0001	rs=0.93		p=0.0001	rs=0.97		p<0.0001
Patient 3	rs=0.81	p=0.0030	rs=0.99		p<0.0001	rs=0.90		p=0.0004	rs=0.95		p<0.0001
Patient 4	rs=0.83	p=0.0024	rs=0.98		p<0.0001	rs=0.93		p=0.0001	rs=0.96		p<0.0001
Patient 5	rs=0.90	p=0.0004	rs=0.98		p<0.0001	rs=0.45		p=0.0956	rs=0.83		p=0.0019

Legend: rs = Spearman’s rank correlation coefficient

The HR and RespRate correlation with the same above mentioned parameters is displayed in table 5. Similar to HR correlation, RMSSD, pNN50 and HF presented a negative correlation with RespRate, respectively LF/HF presented a positive correlation with RespRate.

**Table 5:** Correlation of HR (bpm) and Respiration Rate (breaths/min) with standard HRV parameters

Heart rate (bpm)		RMSSD	pNN50			HF			LF/HF	
Patient 1	rs=-0.63		rs=-0.50		rs=-0.63			rs=0.70		
Patient 2	rs=-0.78		rs=-0.94		rs=-0.65			rs=0.69		
Patient 3	rs=-0.13	p<0.05	rs=-0.21	p<0.05	rs=-0.80		p<0.05	rs=0.80		p<0.05
Patient 4	rs=-0.64		rs=-0.45		rs=-0.46			rs=0.23		
Patient 5	rs=-0.81		rs=-0.44		rs=-0.66			rs=0.63		

RespRate (breaths/min)										
Patient 1	rs=-0.34		rs=-0.71		rs=-0.04			rs=0.32		
Patient 2	rs=-0.90		rs=-0.80		rs=-0.58			rs=0.42		
Pateint 3	rs=-0.72	p<0.05	rs=-0.86	p<0.05	rs=-0.60		p<0.05	rs=0.41		p<0.05
Patient 4	rs=-0.70		rs=-0.61		rs=-0.51			rs=0.16		
Patient 5	rs=-0.97		rs=-0.80		rs=-0.64			rs=0.45		

Legend: rs = Spearman’s rank correlation coefficient, bpm= beats per minute.

**Figure 1:**
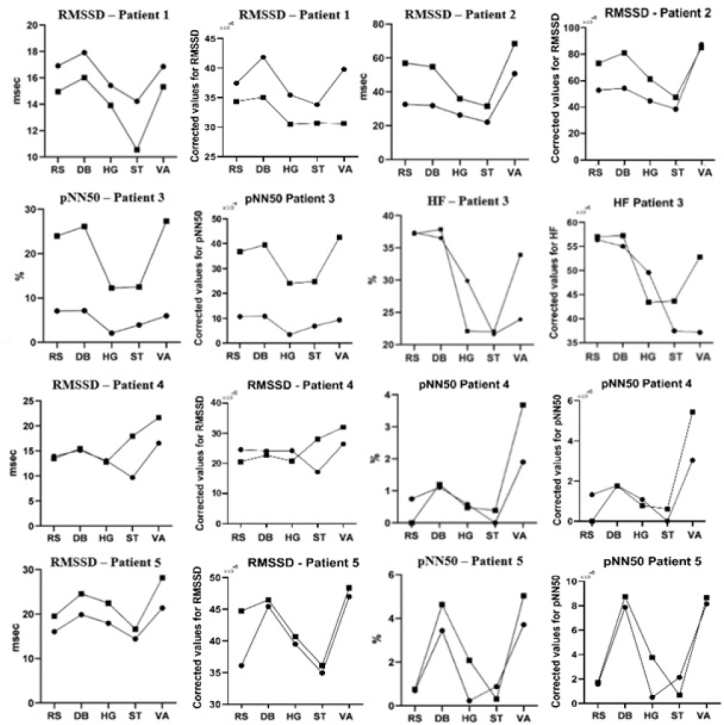
HRV parameters of the five patients. RS=resting state, DB=deep breathing test, HG=hand-grip test, ST=standing test, VA=Valsalva maneuver, 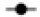
Test 1, 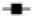
Test 2

The response pattern to autonomic activation tests (deep breathing, hand-grip, standing, Valsalva maneuver) displayed by several HRV parameters was similar after normalization (results illustrated in figure 1).

Appropriate responses to parasympathetic (deep breathing and Valsalva maneuver) and respectively sympathetic (hand-grip and standing) activation tests were observed for all patients, indicated by increases in RMSSD, pNN50 and HF values, respectively decreases of the values of the aforementioned parameters during challenge, in both evaluations.

The first patient presented a slight decrease of RMSSD values after three months of VNS, while the second, the third and the fifth patient displayed an increase of several parasympathetic specific parameters values in the second test. Time and frequency-domain parameters did not reveal a significant change in the cardiac autonomic state after three months of VNS for the fourth patient (figure 1).

**Figure 2:**
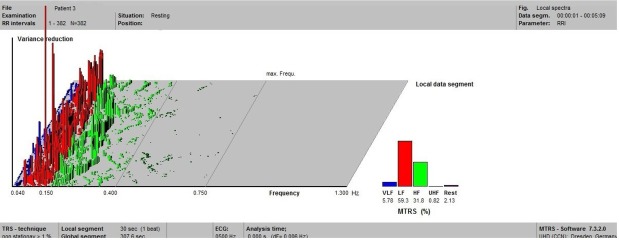
HRV parameters of the third patient in resting state during test 2

**Figure 3:**
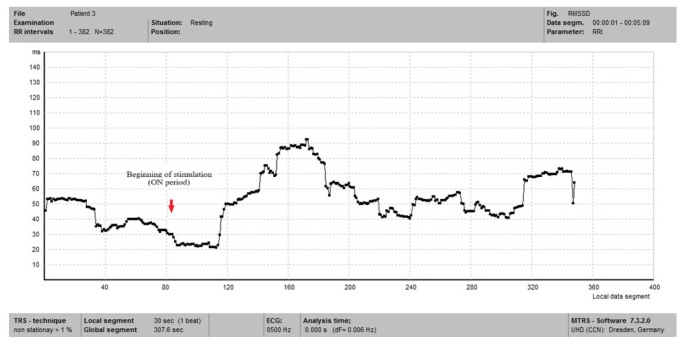
RMSSD values of the third patient in resting state during test 2

The frequency-domain parameters for patient 3 during the second HRV evaluation in resting state are illustrated in figure 2. During the ECG recording, the patient presented the activation of the generator (ON period), being clinically symptomatic (voice alteration and cough). In the interval between seconds 85 and 115 of the ECG recording we identified the ON period, which determined further on an increase of the of RMSSD values (figure 3). This could be an example of the response to left VNS at an intensity sufficient to impose immediate changes in cardiovascular autonomic modulation. In the OFF period, the RMSSD values recovered rapidly to baseline values, with transient overshoot of baseline values (figure 3).

## Discussion

Patients with refractory epilepsy may present decreased HRV, raising the concern that altered autonomic function might contribute to sudden unexpected death in epilepsy (SUDEP) [25, 26]. Long-term recordings in these patients indicated that severe bradycardia or asystole may occur [27, 28], probably related to increased vagal tone associated with sleep. Clinical significance and potential association with the sympathovagal alteration still needs to be clarified. Epileptic seizures involving temporal or insular lobe are susceptible to such complications [28, 29], highlighting the importance of interictal cardiac evaluation in these patients.

The positive effect of VNS in patients with drug-resistant epilepsy is considered to be mediated by the afferent pathways of the vagus nerve, modulating the activity of different cerebral structures, probably involved as trigger-points of seizures [30]. Activation of the vagal efferent pathways may alter the cardiovascular activity, through the sinoatrial node and the cardiac conduction system [30, 31].

All five patients presented normal responses to sympathetic and parasympathetic activation tests in the first HRV evaluation. VNS did not alter HR modulation in response to autonomic activation tests after three months of vagal neuromodulation. This data may indicate the minor contribution to cardiac control of the sympathetic efferent axons contained within the vagosympathetic complex in response to VNS. Cervical vagus nerve is a mixed nerve containing both afferent and efferent axons, belonging to the sympathetic and parasympathetic nervous system [32, 33]. Cardiac-related efferent projections contained within each cervical vagosympathetic trunk are

predominantly parasympathetic preganglionic axons [34]. Parasympathetic preganglionic projections arising bilaterally from nucleus amibiguus innervate multiple intrinsic cardiac ganglionic plexuses located within atrial and ventricular tissues, making direct contact with parasympathetic postganglionic neurons [35]. There is anatomic and functional evidence indicating that the vagosympathetic trunk also contains a small number of sympathetic efferent fibers [35, 36], that modulate cardiac function via the intrinsic cardiac nervous system [37], contributing to the beat-to-beat regulation. Left cervical VNS is believed to minimize potential bradycardia or asystole, primarily mediated by the right vagus nerve [38]. While left cervical VNS is approved for refractory epilepsy and resistant depression, right cervical VNS was clinically tested for heart failure [38].

Chronic VNS influences both sympathetic and parasympathetic modulation but does not negatively influence autonomic cardiovascular regulation [39, 40]. Increasing parasympathetic

activity seems to have cardioprotective effects against SUDEP [41]. Cardiac bradyarrhythmia represents a rare complication, few isolated cases during ongoing VNS therapy being reported [42, 43, 44, 45, 46]. VNS may also enhance sympathetic output, raising hippocampal noradrenaline levels [9, 47]. Locus coeruleus, the principal noradrenergic nucleus of the brain, is involved in the circuitry necessary for the anticonvulsant effectiveness of VNS [48]. Seizure suppression by VNS in drug-resistant epilepsy patients may, therefore, depend on the release of noradrenaline, a neuromodulator that has anticonvulsant effects.

Another cortical structure involved in autonomic regulation is insula. Insular lesions are associated with mortality through autonomic dysfunction [29]. Electrical stimulation of the human insula produced cardiac chronotropic and blood pressure responses in epilepsy patients [49]. Right insula seems to regulate the sympathetic tone, while left insula is associated with prevalent parasympathetic control [50]. There is contradicting evidence supporting cortical lateralization of autonomic control [51, 52]. Four of our five patients had insular or temporal epilepsy. Patient 3, with left insular epilepsy, and patient 5, with right insular epilepsy, presented both appropriate responses to autonomic activation tests and a slight increase in several parasympathetic specific parameters after three months of VNS.

Antiepileptic drugs can also influence autonomic activity. Carbamazepine has been shown to affect the autonomic modulation

among patients with temporal lobe epilepsy [53, 54], and withdrawal can increase cardiac sympathetic activity during sleep [55]. Patient 4 was under Carbamazepine and HRV analysis indicated a predominance of the sympathetic tonus in both evaluations, requiring further cardiac monitoring. Lamotrigine is supposed to have cardiac arrhythmogenic potential in certain metabolic conditions. Further research is needed to analyze the clinical impact [56].

The strengths of our report include MTRS analysis of the HRV on short ECG recordings (5 minutes) after autonomic activation tests. The advantage of MTRS analysis, which does not need interpolation on non-equidistant RR intervals contrary to Fast Fourier Transformation, is the assessment of shorter local data segments [11, 13]. An adequate correction designed to remove the HR influence on HRV should always be performed, due to physiological but also mathematical reasons [17, 18, 19].

One of the limits of our report is the reduced number of patients while the other is using surface EEG study, because of the distant location of the insular cortex relative to scalp electrodes and the rapidly spreading activity.

Autonomic nervous system should be evaluated in epilepsy patients because of the risk of cardiac arrhythmias and SUDEP, particularly in the drug-resistant ones. We propose HRV analysis as a useful tool to assess sympathovagal balance and identify high-risk patients for cardiac arrhythmias. Moreover, HRV analysis could be a practical tool in identifying suitable patients for VNS therapy.

## References

[1] Moshé SL, Perucca E, Ryvlin R, Tomson T (2015). Epilepsy: new advances.

[2] Devinsky O, Hesdorffer DC, Thurman DJ, Lhatoo S, Richersonet G (2016). Sudden unexpected death in epilepsy: epidemiology, mechanisms, and prevention. Lancet Neurol.

[3] Harden C, Tomson T, Gloss D, Buchhalter J, Cross JH, Donner E (2017). Practice guideline summary: Sudden unexpected death in epilepsy incidence rates and risk factors: Report of the Guideline Development, Dissemination, and Implementation Subcommittee of the American Academy of Neurology and the American Epilepsy Society. Neurology.

[4] Kwan P, Arzimanoglou A, Berg AT, Brodie MJ, Hauser WA, Mathernet G (2010). Definition of drug-resistant epilepsy: consensus proposal by the ad hoc Task Force of the ILAE Commission on Therapeutic Strategies. Epilepsia.

[5] Panebianco M, Zavanone C, Dupont S, Restivo DA, Pavone A (2016). Vagus nerve stimulation therapy in partial epilepsy: a review. Acta Neurol Belg.

[6] Morris GL, Gloss D, Buchhalter J, Mack KJ, Nickels K, Harden C (2013). Evidence-based guideline update: Vagus nerve stimulation for the treatment of epilepsy. Neurology.

[7] Ryvlin P, Gilliam FG, Nguyen DK, Colicchio G, Iudice A, Tinuperet P (2014). The long-term effect of vagus nerve stimulation on quality of life in patients with pharmacoresistant focal epilepsy: the PuLsE (Open Prospective Randomized Long-term Effectiveness) trial. Epilepsia.

[8] Fornai F, Ruffoli R, Giorgi FS, Paparelli A (2011). The role of locus coeruleus in the antiepileptic activity induced by vagus nerve stimulation. Eur J Neurosci.

[9] Garamendi I, Acera M, Agundez M, Galbarriatu L, Marinas A, Pomposo I (2017). Cardiovascular autonomic and hemodynamic responses to vagus nerve stimulation in drug-resistant epilepsy. Seizure.

[10] (1996). Task Force of the European Society of Cardiology and the North American Society of Pacing and Electrophysiology. Heart rate variability: standards of measurement, physiological interpretation, and clinical use. Circulation.

[11] Rüdiger H, Klinghammer L, Scheuch K (1999). The trigonometric regressive spectral analysis--a method for mapping of beat-to-beat recorded cardiovascular parameters on to frequency domain in comparison with Fourier transformation. Comput Methods Programs Biomed.

[12] Myers KA, Bello-Espinosa LE, Symonds JD, Zuberi SM, Clegg R, Sadleir LG (2018). Heart rate variability in epilepsy: A potential biomarker of sudden unexpected death in epilepsy risk. Epilepsia.

[13] Li K, Rüdiger H, Haase R, Ziemssen T (2018). An Innovative Technique to Assess Spontaneous Baroreflex Sensitivity with Short Data Segments: Multiple Trigonometric Regressive Spectral Analysis. Front Physiol.

[14] Billman GE (2013). The LF/HF ratio does not accurately measure cardiac sympatho-vagal balance. Front Physiol.

[15] Moak JP, Goldstein DS, Eldadah BA, Saleem A, Holmes C, Pechnik S (2007). Supine low-frequency power of heart rate variability reflects baroreflex function, not cardiac sympathetic innervation. Heart Rhythm.

[16] Gąsior JS, Sacha J, Pawłowski M, Zieliński J, Jeleń PJ, Tomik A (2018). Normative Values for Heart Rate Variability Parameters in School-Aged Children: Simple Approach Considering Differences in Average Heart Rate. Front. Physiol.

[17] Sacha J, Pluta W (2008). Alterations of an average heart rate change heart rate variability due to mathematical reasons. Int. J. Cardiol.

[18] Sacha J, Pluta W (2005). Different methods of heart rate variability analysis reveal different correlations of heart rate variability spectrum with average heart rate. J. Electrocardiol.

[19] Sacha J, Barabach S, Statkiewicz-Barabach G, Sacha K, Muller A, Piskorski J (2013). How to strengthen or weaken the HRV dependence on heart rate—Description of the method and its perspectives. Int. J. Cardiol.

[20] Monfredi O, Lyashkov AE, Johnsen AB, Inada S, Schneider H, Wang R (2014). Biophysical characterization of the underappreciated and important relationship between heart rate variability and heart rate. Hypertension.

[21] Gąsior JS, Sacha J, Jeleń PJ, Zieliński J, Przybylski J. Heart Rate, Respiratory Rate (2016). Influence on Heart Rate Variability Repeatability: Effects of the Correction for the Prevailing Heart Rate. Front Physiol.

[22] Bruce EN (1996). Temporal variations in the pattern of breathing. J Appl Physiol.

[23] Quintana DS, Heathers JA (2014). Considerations in the assessment of heart rate variability in biobehavioral research. Front Psychol.

[24] Sinnecker D, Dommasch M, Barthel P, Müller A, Dirschinger RJ, Hapfelmeier A (2014). Assessment of mean respiratory rate from ECG recordings for risk stratification after myocardial infarction. J Electrocardiol.

[25] Massetani R, Strata G, Galli R, Gori S, Gneri C, Limbruno U (1997). Alteration of cardiac function in patients with temporal lobe epilepsy: different roles of EEG-ECG monitoring and spectral analysis of RR variability. Epilepsia.

[26] Ansakorpi H, Korpelainen JT, Huikuri HV, Tolonen U, Myllylä VV, Isojärvi JI (2002). Heart rate dynamics in refractory and well controlled temporal lobe epilepsy. J Neurol Neurosurg Psychiatry.

[27] Nei M (2009). Cardiac effects of seizures. Epilepsy Curr.

[28] Rugg-Gunn F, Simister RJ, Squirell M, Holdright DR, Duncan JS (2004). Cardiac arrhythmias in focal epilepsy: a prospective long-term study. Lancet.

[29] Lacuey N, Zonjy B, Theerannaew W, Loparo KA, Tatsuoka C, Sahadevan J (2016). Left-insular damage, autonomic instability, and sudden unexpected death in epilepsy. Epilepsy Behav.

[30] Henry TR (2002). Therapeutic mechanisms of vagus nerve stimulation. Neurology.

[31] Premchand RK, Sharma K, Mittal S, Monteiro R, Dixit S, Libbus I (2016). Extended Follow-Up of Patients with Heart Failure Receiving Autonomic Regulation Therapy in the ANTHEM-HF Study. J Card Fail.

[32] Bonaz B, Picq C, Sinniger V, Mayol JF, Clarencon D (2013). Vagus nerve stimulation: from epilepsy to the cholinergic anti-inflammatory pathway. Neurogastroenterol Motil.

[33] Hoover DB, Shepherd AV, Southerland EM, Armour JA, Ardell JL (2008). Neurochemical diversity of afferent neurons that transduce sensory signals from dog ventricular myocardium. Auton Neurosci.

[34] Randall WC, Ardell JL, Becker DM (1985). Differential responses accompanying sequential stimulation and ablation of vagal branches to dog heart. Am J Physiol Heart Circ Physiol.

[35] Ardell JL, Rajendran PS, Nier HA, KenKnight BH, Armour JA (2015). Central-peripheral neural network interactions evoked by vagus nerve stimulation: functional consequences on control of cardiac function. Am J Physiol Heart Circ Physiol.

[36] Onkka P, Maskoun W, Rhee KS, Hellyer J, Patel J, Tan J (2013). Sympathetic nerve fibers and ganglia in canine cervical vagus nerves: localization and quantitation. Heart Rhythm.

[37] Randall DC, Brown DR, McGuirt AS, Thompson GW, Armour JA, Ardell JL (2003). Interactions within the intrinsic cardiac nervous system contribute to chronotropic regulation. Am J Physiol Regul Integr Comp Physiol.

[38] (2014). Howland RH. Vagus Nerve Stimulation. Curr Behav Neurosci Rep.

[39] Stemper B, Devinsky O, Haendl T, Welsch G, Hilz MJ (2008). Effects of vagus nerve stimulation on cardiovascular regulation in patients with epilepsy. Acta Neurol Scand.

[40] Ronkainen E, Korpelainen JT, Heikkinen E, Myllylä VV, Huikuri HV, Isojärvi JI (2006). Cardiac autonomic control in patients with refractory epilepsy before and during vagus nerve stimulation treatment: a one-year follow-up study. Epilepsia.

[41] Schomer AC, Nearing BD, Schachter SC, Verrier RL (2014). Vagus nerve stimulation reduces cardiac electrical instability assessed by quantitative T-wave alternans analysis in patients with drug-resistant focal epilepsy. Epilepsia.

[42] Asconape JJ, Moore DD, Zipes DP, Hartman LM, Duffell Jr WH (1999). Bradycardia and asystole with the use of vagus nerve stimulation for the treatment of epilepsy: a rare complication of intraoperative device testing. Epilepsia.

[43] Tatum WO, Moore DB, Stecker MM, Baltuch GH, French JA, Ferreira JA (1999). Ventricular asystole during vagus nerve stimulation for epilepsy in humans. Neurology.

[44] Ali II, Pirzada NA, Kanjwal Y, Wannamaker B, Medhkour A, Koltz MT (2004). Complete heart block with ventricular asystole during left vagus nerve stimulation for epilepsy. Epilepsy Behav.

[45] Iriarte J, Urrestarazu E, Alegre M, Macías A, Gómez A, Amaro P (2009). Late-onset periodic asystolia during vagus nerve stimulation. Epilepsia.

[46] Amark P, Stodberg T, Wallstedt L. (2007). Late onset bradyarrhythmia during vagus nerve stimulation. Epilepsia.

[47] Raedt R, Clinckers R, Mollet L, Vonck K, El Tahry R, Wyckhuyset T (2011). Increased hippocampal noradrenaline is a biomarker for efficacy of vagus nerve stimulation in a limbic seizure model. J Neurochem.

[48] Krahl SE, Clark KB, Smith DC, Browning RA (1998). Locus coeruleus lesions suppress the seizureattenuating effects of vagus nerve stimulation. Epilepsia.

[49] Oppenheimer S, Hachinski V (1992). Complications of acute stroke. Lancet.

[50] Oppenheimer SM, Gelb A, Girvin JP, Hachinscki VC (1992). Cardiovascular effects of human insular cortex stimulation. Neurology.

[51] Constantinescu V, Matei D, Cuciureanu D, Corciova C, Ignat B, Popescu CD (2016). Cortical modulation of cardiac autonomic activity in ischemic stroke patients. Acta Neurol Belg.

[52] Di Gennaro G, Quarato PP, Sebastiano F, Esposito V, Onorati P, Grammaldo LG (2004). Ictal heart rate increase precedes EEG discharge in drug-resistant mesial temporal lobe seizures. Clin Neurophys.

[53] Isojarvi JIT, Ansakorpi H, Suominen K, Tolonen U, Repo M, Myllylä VV (1998). Interictal cardiovascular autonomic responses in patients with epilepsy. Epilepsia.

[54] Ansakorpi H, Korpelainen JT, Suominen K, Tolonen U, Myllylä VV, Isojärvi JI (2000). Interictal cardiovascular autonomic responses in patients with temporal lobe epilepsy. Epilepsia.

[55] Hennessy MJ, Tighe MG, Binnie CD, Nashef L (2001). Sudden withdrawal of carbamazepine increases cardiac sympathetic activity in sleep. Neurology.

[56] Daniellson BR, Lansdell K, Patmore L, Tomson T (2005). Effects of the antiepileptic drugs lamotrigine, topiramate and gabapentin on hERG potassium currents. Epilepsy Res.

